# Association of adverse childhood experiences with health service use and catastrophic health expenditures in China: evidence from the China Health and Retirement Longitudinal Study

**DOI:** 10.1265/ehpm.25-00012

**Published:** 2026-01-01

**Authors:** Shiyu Xie, Siying Yu, Yue Ma, Jing Luo, Yonghui Zhang, Rui Wang, Yafei Wang, Yuling Wang, Xueqiang Wang

**Affiliations:** 1 Department of Basic Teaching, Shanghai Urban Construction Vocational College, Shanghai, 201415, China; 2 Department of Cardiology, Fuwai Shenzhen Hospital, Chinese Academy of Medical Sciences, Shenzhen, 518052, China; 3 Rehabilitation Medicine Center, The Second Affiliated Hospital of Wenzhou Medical University, Wenzhou, 325027, China; 4 Department of Rehabilitation Medicine, The Sixth Affiliated Hospital of Sun Yat-sen University, Guangzhou, 510655, China; 5 Guangdong Provincial Clinical Research Center for Rehabilitation Medicine, Guangzhou, 510655, China; 6 Biomedical Innovation Center, The Sixth Affiliated Hospital, SunYat-sen University, Guangzhou, 510655, China; 7 Department of Sport Rehabilitation, Xi’an Physical Education University, Xi’an, 710068, China

**Keywords:** Adverse childhood experiences, Catastrophic health expenditures, Child abuse, Epidemiology, Financial stresses, Mediation

## Abstract

**Backgrounds:**

Associations between adverse childhood experiences (ACEs) and catastrophic health expenditures (CHEs) among middle-aged or older Chinese individuals remain inadequately documented. In addition, the role of chronic diseases is not entirely clear. This study used data from the China Health and Retirement Longitudinal Study (CHARLS) to investigate the association of ACEs with hospital visits and medical expenditures and the mediating effect of chronic diseases.

**Methods:**

Data were collected from CHARLS in 2014 and 2015 (N = 11,072). Zero-inflated negative binomial regression was used to assess associations of the ACEs with the number of outpatient visits and inpatient hospital days. Logistic regression was used to assess associations between the ACEs and CHEs. The influence of chronic diseases was examined through mediation analysis.

**Results:**

The prevalence of each ACE indicator ranges from 0.27% (incarcerated household member) to 31.5% (emotional neglect). Our analysis revealed a significant dose-response relationship between cumulative ACE score and CHEs (P for trend < 0.001), but not for the number of outpatient visits and inpatient hospital days. The average causal mediation effects (ACME) and average direct effects (ADE) are presented. Chronic diseases served as a mediating factor between ACEs and CHE (ACME = 0.000904, P = 0.03; ADE = 0.00813, P < 0.001).

**Conclusions:**

ACE has the capacity to predict CHE, and the findings of this study reinforce the potential pathway through which ACE may exert its influence on CHE via the burden of chronic diseases. Measures should be implemented to prevent ACEs and mitigate the risk of chronic diseases to lessen the economic burden on individuals and families as well as the adverse impact of national financial risk.

**Supplementary information:**

The online version contains supplementary material available at https://doi.org/10.1265/ehpm.25-00012.

## Introduction

Adverse childhood experiences (ACEs) are potentially traumatic events that occur during youth. These events are classified into two categories: those that directly affect children (e.g., physical abuse and emotional neglect) and those that indirectly affect children through their environment (e.g., family mental illness and parental separation or divorce) [1]. The high prevalence of ACEs has prompted researchers to focus more closely on their effects [2, 3]. Many studies suggest that ACEs are associated with several long-term adverse health outcomes [4], including dementia [5], cardiovascular diseases [6], and mental health problems, such as borderline personality disorder and anxiety [7, 8]. These psychological and physical health issues are likely to increase the overall cost of healthcare, thereby contributing to a greater healthcare burden. Consequently, the direct and indirect financial burden associated with ACEs highlights the need to focus on ACEs’ associations with healthcare services and expenditures. This is further illustrated by the substantial economic burden of ACE-related health problems in developed countries in recent years [3].

Most studies exploring the connection between ACEs and medical burdens have primarily focused on women and children in Europe and America who experienced childhood abuse, as well as the short- and long-term social and healthcare costs associated with ACEs [9–11]. However, there is a paucity of research on the relationships between the 12 types of ACEs and medical behaviors, including both outpatient and inpatient services, particularly in developing countries such as China. As mentioned earlier, ACEs are associated with many chronic diseases. Nevertheless, whether ACEs influence healthcare utilization by affecting health outcomes is still unclear. Based on previous studies, we conducted a mediation analysis by using chronic diseases as a potential pathway through which ACEs influence healthcare use.

This study utilizes data from the China Health and Retirement Longitudinal Study (CHARLS), a continuous nationwide representative longitudinal survey, to analyze 12 indicators of ACEs among Chinese adults. CHARLS collected high-quality data from individuals aged 45 and older through one-on-one interviews and structured questionnaires. We explored the association between ACEs and medical behaviors, including outpatient visits, inpatient hospital days, and catastrophic health expenditures (CHEs), while examining the mediating effects of chronic diseases. This research aims to enhance understanding of the medical behaviors and economic burdens associated with ACEs, thereby drawing greater societal attention to the physical and mental health impacts on children.

## Methods and materials

### Population

**Fig. 1:**
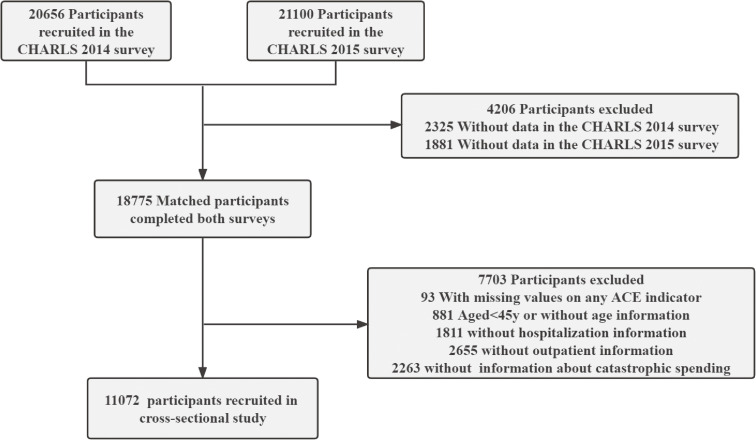
Flowchart of of 11,072 Chinese adults in the 2015 CHARLS study.

This cross-sectional study utilized data from the CHARLS. Detailed descriptions of the study design and sampling methods have been previously documented in the literature [12]. Briefly, participants in the CHARLS were selected through a multistage probability sampling strategy. The baseline survey included 17,708 participants from 450 villages or residential communities across 28 provinces in China. Participants were followed up every two years, with a small number of new individuals added in each subsequent survey. The Biomedical Ethics Committee of Peking University approved the CHARLS survey project, and all participants were required to provide informed consent by signing documents. To date, five follow-up surveys have been conducted in 2011, 2013, 2015, 2018, and 2020, respectively. Information regarding childhood experiences was collected during the 2014 Life History Survey, which was administered to all surviving respondents from the 2011 and 2013 surveys. The current analysis was based on data from the 2014 Life History Survey (conducted from June 1 to December 31, 2014) and the 2015 follow-up survey (conducted from July 1 to September 30, 2015), when the most recent health assessment data were available. A total of 20,656 and 21,100 individuals participated in the CHARLS, respectively (Fig. 1). We conducted an initial screening of 18,775 respondents who completed two rounds of surveys. After excluding 93 participants with missing values for any of the ACE indicators, 881 individuals under 45 years old or without age information, 1,811 individuals lacking hospitalization information, 2,655 individuals without outpatient information, and 2,263 without information about catastrophic spending, a sample of 11,072 individuals remained for data analysis. This study followed the Strengthening the Reporting of Observational Studies in Epidemiology reporting guideline.

### Definition of ACEs

Referring to previous studies on ACEs, we extracted 12 ACEs from our dataset [4] (e.g., parental death, parental separation or divorce, sibling death, bullying, household mental illness, parental disability, household substance abuse, emotional neglect, physical abuse, domestic violence, incarcerated household member, and unsafe neighborhood) (Table S1). Furthermore, the cumulative score was calculated based on the values of 0, 1, 2, 3, and ≥4.

### Calculation methods for the number of outpatient visits, number of inpatient hospital days, and CHEs

The World Health Organization proposes defining CHE as out-of-pocket health expenditures equal to or exceeding 40% of a household’s ability to pay [13]. We define the payment capacity of a family as the total consumption expenditure of the family minus the consumption expenditure related to food. Annual out-of-pocket health expenditures include the total costs of outpatient care and hospitalization during the previous year. The 2015 questionnaire collected data on respondents’ out-of-pocket outpatient expenses in the past month, which we multiplied by 12 to estimate a year’s outpatient expenses. Hospitalization costs were calculated from the total out-of-pocket costs reported by respondents in the past year. The number of outpatients in the past year was calculated from the sum of outpatients in the past month answered by the respondents. Hospitalizations were recorded based on responses to the question: “How many times have you received inpatient care during the past year?”.

### Covariates

We considered the following participant characteristics: age, sex (female or male), marital status (married or unmarried), education level (none, homeschooled, primary school, middle school, and above), smoking status (current, former, and never smoked), drinking habits (never drink, less than once a month, and more than once a month), place of residence (rural or urban), number of chronic diseases (0, 1, 2, ≥3), economic development region (Groups 1–5), socioeconomic group (Quartiles 1–4), health insurance (none, Urban Employee Basic Medical Insurance, Urban Resident Basic Medical Insurance, New Rural Cooperative Medical Scheme, and others), and sleep duration. According to the question-and-answer content regarding “Have you been diagnosed with [the conditions listed below, read one by one] by a doctor?”, calculate the chronic diseases mainly includes the following: hypertension, dyslipidemia, diabetes, cancer, chronic lung diseases, liver disease, heart problems, stroke, kidney disease, digestive diseases, arthritis, asthma, psychiatric diseases and memory-related diseases.

Socioeconomic status is defined by calculating the quartile of per capita consumption for the entire previous year. Five economic development regions were designated according to the ranking of China’s provincial gross domestic product per capita in 2015 (Group 1, >$12,000; Group 2, $12,000 to >$10,000; Group 3, $10,000 to >$7,000; Group 4, $7,000 to >$6,000; and Group 5, ≤$6,000). These covariates were selected due to their potential influence on health outcomes and healthcare utilization, providing a comprehensive understanding of the factors affecting the associations under investigation [14–16]. Detailed information about the covariates is provided in Table S2.

### Statistical analysis

Continuous variables are reported as means and standard deviations (SDs), and categorical variables are expressed as counts and percentages. The Kruskal–Wallis H test and χ^2^ test were used to compare the characteristics of continuous and categorical variables between different groups, respectively.

Given the highly dispersed nature of the number of outpatient visits and inpatient hospital days, we employed the negative binomial regression model instead of the Poisson regression model to investigate the association between ACEs and the number of outpatient visits as well as inpatient hospital days. Subsequently, based on the results of the vuong tests, we finally used the zero-inflated negative binomial regression. Additionally, we employed logistic regression models to estimate the relationship between the number of ACEs and CHE.

For logistic regression and negative binomial regression, we reported the odds ratios (ORs) and 95% confidence intervals (CIs), and the adjusted incidence rate ratios (IRRs) and 95% CIs, respectively. Five models were estimated: Model 1 was the crude model; Model 2 was adjusted for age, sex, marital status, area of residence, education, and sleep duration; Model 3 was adjusted for age, sex, marital status, area of residence, education, sleep duration, smoking status, and drinking status; Model 4 was adjusted for age, sex, marital status, area of residence, education, sleep duration, smoking status, drinking status, and chronic diseases; and Model 5 was adjusted for age, sex, marital status, area of residence, education, sleep duration, smoking status, drinking status, chronic diseases, socioeconomic status, health insurance, and economic development regions. We primarily evaluated the dose-response relationship between ACEs and three outcomes [4, 17]. Each subsequent model built upon the previous one, progressively examined the influence of other covariates on the relationship between ACEs and outcomes. We contrasted models using likelihood ratio tests [18]. In the sensitivity analysis section, we used the truncated method at the 95th percentile to handle the annual outpatient expenses and then conducted a logistic regression again.

We employed the Multiple Imputation by Chained Equations method, which accounts for variability between samples and generates multiple imputed datasets to better reflect uncertainty in the data [19] (Table S3). By analyzing each imputed dataset and combining the results, we obtained more robust estimates of the relationships under investigation. Additionally, we conducted stratified analyses by age and gender to explore differences among age groups and genders.

To further explore the mediating effect of chronic diseases on the relationships between ACEs and the number of outpatient visits, inpatient hospital days, and CHE, we constructed two regression models for each outcome. One was a ordered logistic regression of the mediating variable (chronic diseases) and independent variable (ACEs), and the other was a regression of dependent variables (number of outpatient visits, inpatient hospital days, and CHE), the independent variable (ACEs) and the mediating variable (chronic diseases). All regression models were adjusted for covariates. Bootstrap methods were then employed to test the mediation effect [20]. We further conducted a mediation analysis using the presence or absence of chronic diseases as a binary variable. The mediation analysis relies on the strong assumption of sequential ignorability, which requires that there are no unmeasured confounding variables affecting (a) the mediator-outcome relationship and (b) the treatment-mediator-outcome relationships. We conducted a sensitivity analysis (medsens) to quantify the robustness of our findings to potential violations of this assumption.

Statistical analyses were conducted using R version 4.3.1, with the mice package for multiple imputation and the mediation package for mediation analysis [21]. A significance level of 0.05 was set for all the statistical tests.

## Results

The baseline characteristics of the 2015 study population are presented in Table 1. The average age of the 11,072 individuals was 59.64 years (SD = 9.68 years), 49.70% of the participants were male, 83.78% were married, 75.12% lived in rural areas, 53.62% had no or only primary education, 32.96% were smokers, 56.40% had never consumed alcohol, 35.27% reported no chronic diseases, and 14.52% reported at least three chronic diseases. Furthermore, as the cumulative ACE score increased, the proportion of participants who were unmarried, lived in rural areas, regularly consumed alcohol, and had chronic diseases also increased. We observed that the reduction in sleep duration was associated with the accumulation of ACEs. The prevalence of each ACE indicator varied from 0.27% (incarcerated household member) to 31.5% (emotional neglect) (Table S4). Moreover, 83.78% of the respondents had purchased at least one type of medical insurance, and 70.90% were enrolled in the New Rural Cooperative Medical Scheme. The proportion of medical care utilization significantly increased with age. Among individuals aged 45–59 years, 7% had at least one outpatient visit, 3% had at least one day of hospitalization, and 7% had experienced CHE. Among those aged 60 years and older, 11% had at least one outpatient visit, 5% had been hospitalized, and 11% had CHE (Fig. S1).

**Table 1:** Characteristics of 11,072 Chinese adults from CHARLS study 2015.

**ACEs (N = 11072)**						

	**ACE = 0** **(n = 2748)**	**ACE = 1** **(n = 3484)**	**ACE = 2** **(n = 2479)**	**ACE = 3** **(n = 1396)**	**ACE ≥ 4** **(n = 965)**	**p-value**
**Ages, year**	59.63(9.85)	59.88(9.72)	59.56 (9.71)	59.42 (9.45)	59.26(9.31)	0.387*
**Sex**						
Male	1231(44.80%)	1769(50.77%)	1287 (51.92%)	737(52.79%)	479(49.64%)	<0.001^†^
Female	1517(55.20%)	1715(49.23%)	1192 (48.08%)	659 (47.21%)	486(50.36%)	

**Marital status**						
Married	2363(85.99%)	2931(84.02%)	2061 (83.14%)	1139 (81.59%)	782(81.04%)	<0.001^†^
Unmarried	385(14.01%)	553(15.87%)	418 (16.86%)	257 (18.41%)	183(18.96%)	

**Residence**						
Rural	2032(73.94%)	2583(74.14%)	1875 (75.63%)	1084 (77.65%)	748(77.51%)	0.01^†^
Urban	712(25.91%)	888(25.49%)	600 (24.20%)	305 (21.85%)	213(22.49%)	

**Educational level**						
None	539(19.61%)	621(17.828%)	438 (17.67%)	284 (20.34%)	200(20.73%)	<0.001^†^
Home school to primary school	974(35.44%)	1366(39.21%)	1012 (40.82%)	609 (43.62%)	434(44.97%)	
Middle school or above	1232(44.83%)	1497(42.97%)	1029(41.51%)	503 (36.03%)	331(34.30%)	

**Smoking status**						
Smoker	728 (26.49%)	1043 (29.94%)	799 (32.23%)	450 (32.23%)	302(31.30%)	0.24^†^
Ever smoker	335 (12.19%)	454 (13.03%)	315 (12.71%)	162 (11.60%)	113(11.71%)	
Never smoking	52 (1.89%)	91 (2.61%)	64 (2.58%)	43 (3.08%)	26(2.69%)	

**Drinking status**						
Never drinking	1660 (60.41%)	1939 (55.65%)	1287 (51.91%)	728 (52.15%)	491(50.88%)	<0.001^†^
Less than once a month	200 (7.28%)	271 (7.78%)	223 (8.99%)	129 (9.24%)	75(7.77%)	
More than once a month	220 (8.01%)	282 (8.09%)	209 (8.43%)	129 (9.24%)	106(10.98%)	

**Chronic diseases**						
0	1066 (38.79%)	1253 (35.96%)	845 (34.09%)	442 (31.66%)	299(30.98%)	<0.001^†^
1	842 (30.64%)	1129 (32.41%)	820 (33.08%)	478 (34.24%)	314(32.54%)	
2	487 (17.72%)	596 (17.11%)	439 (17.71%)	261 (18.7%)	193(20.00%)	
≥3	353 (12.85%)	506 (14.52%)	375 (15.13%)	215 (15.4%)	159(16.84%)	

**Sleep duration**	6.26(2.37)	6.22(2.19)	6.14(2.24)	6.07(2.28)	5.85(2.25)	<0.001*

**Economic development region**						
Group 1 (most affluent)	270 (9.83%)	404 (11.6%)	311 (12.55%)	200 (14.33%)	132(13.68%)	<0.001^†^
Group 2	806 (29.33%)	966 (27.73%)	683 (27.55%)	367 (26.29%)	233(24.15%)	
Group 3	175 (6.37%)	290 (8.32%)	216 (8.71%)	164 (11.75%)	120(12.44%)	
Group 4	787 (28.64%)	994 (28.53%)	687 (27.71%)	378 (27.08%)	285(29.53%)	
Group 5	710 (25.84%)	830 (23.82%)	582 (23.48%)	287 (20.56%)	195(20.21%)	

**Socioeconomic group**						
Quartile 1 (highest)	712 (25.91%)	859 (24.66%)	629 (25.37%)	345 (24.71%)	246(25.49%)	0.33^†^
Quartile 2	699 (25.44%)	931 (26.72%)	603 (24.32%)	365 (26.15%)	267(27.67%)	
Quartile 3	657 (23.91%)	827 (23.74%)	647 (26.1%)	334 (23.93%)	207(21.45%)	
Quartile 4 (lowest)	680 (24.75%)	867 (24.89%)	600 (24.2%)	352 (25.21%)	245(25.39%)	

**Health insurance**						
None	299 (10.88%)	349 (10.02%)	250 (10.08%)	167 (11.96%)	103(10.67%)	<0.001^†^
UEBMI	327 (11.9%)	430 (12.34%)	277 (11.17%)	122 (8.74%)	70(7.25%)	
URBMI	125 (4.55%)	160 (4.59%)	94 (3.79%)	64 (4.58%)	36(3.37%)	
NRCMS	1914 (69.65%)	2429 (69.72%)	1782 (71.88%)	1005 (71.99%)	728(75.44%)	
Others	83 (3.02%)	116 (3.33%)	76 (3.07%)	38 (2.72%)	28(2.90%)	

Note: ACE, adverse childhood experience; UEBMI, Urban Employee Basic Medical Insurance; URBMI, Urban Resident Basic Medical Insurance; NRCMS, New Rural Cooperative Medical Scheme. A Continuous data are reported as the mean (SD), and categorical data are reported as the number and percentage of participants. The *Kruskal–Wallis H test and ^†^χ^2^ test were used to compare the characteristics of ACEs among different groups for continuous and categorical variables, respectively.

We found that individuals who experienced two, three, and four ACEs had 26% (OR = 1.26, 95% CI: 1.03–1.54), 34% (OR = 1.34, 95% CI: 1.06–1.69), and 73% (OR = 1.73, 95% CI: 1.35–2.21) increased likelihood of experiencing CHE, respectively, compared with those who experienced no ACEs, respectively (Model 2 and 5 are presented in Table 2, while Model 1, 3, and 4 are shown in Table S5). Increasing ACEs were not significantly associated with an increased risk of outpatient visits or inpatient hospital days Table 3. Poisson regression analyses and other imputed data yielded similar results (Table S6 and Table S7).

**Table 2:** The association between adverse childhood experiences and catastrophic health expenditure

	**ACE = 0**	**ACE = 1**	**ACE = 2**	**ACE = 3**	**ACE ≥ 4**	**P for trend**

		**IRR/OR(95%CI)**	**IRR/OR(95%CI)**	**IRR/OR(95%CI)**	**IRR/OR(95%CI)**	
Model 2^†^						
CHE	Reference	1.11(0.92,1.34)	1.33(1.09,1.63)**	1.45(1.16,1.82)**	1.91(1.50,2.42)***	<0.001
Model 5^∥^						
CHE	Reference	1.06(0.87,1.28)	1.26(1.03,1.54)*	1.34(1.06,1.69)*	1.73(1.35,2.20)***	<0.001
Sensitivity analysis^§^						
CHE	Reference	1.06(0.87–1.28)	1.27(1.04–1.56)*	1.39(1.10–1.75)**	1.80(1.41–2.30)***	<0.001

Note: CHE, catastrophic health expenditure

^†^Model 2 was adjusted for age, sex, marital status, area of residence, education, sleeping time.

^∥^Model 5 was adjusted for age, sex, marital status, area of residence, education, sleeping time, smoke status, drink status, chronic diseases, socioeconomic status, health insurance, and economic development regions.

^§^we used the truncated method at the 95th percentile to handle the annual outpatient expenses and then conducted a logistic regression again.

*p < 0.05, **p < 0.01, ***p < 0.001

**Table 3:** The association between adverse childhood experiences and the number of outpatient visits and inpatient hospital days.

	**ACE = 0**	**ACE = 1**	**ACE = 2**	**ACE = 3**	**ACE ≥ 4**

		**IRR/OR(95%CI)**	**IRR/OR(95%CI)**	**IRR/OR(95%CI)**	**IRR/OR(95%CI)**
Model 2^†^					
Number of outpatient visits (count)	Reference	0.99(0.89–1.11)	0.86(0.77–0.97)	0.89(0.78–1.03)	0.85(0.73–0.99)
Number of outpatient visits (zero)	Reference	1.02(0.87–1.19)	1.05(0.88–1.25)	0.98(0.80–1.20)	0.96(0.76–1.21)
Inpatient hospital days (count)	Reference	0.99(0.81–1.23)	0.87(0.70–1.10)	1.01(0.78–1.32)	1.02(0.79–1.31)
Inpatient hospital days (zero)	Reference	0.87(0.67–1.15)	0.78(0.58–1.05)	0.92(0.64–1.32)	0.52(0.36–0.73)
Model 5^∥^					
Number of outpatient visits count	Reference	0.99(0.89–1.10)	0.89(0.79–0.99)	0.92(0.80–1.06)	0.89(0.76–1.04)
Number of outpatient visits zero	Reference	1.01(0.86–1.18)	1.04(0.88–1.24)	0.97(0.79–1.19)	0.96(0.76–1.21)
Inpatient hospital days count	Reference	0.97(0.87–1.21)	0.84(0.67–1.07)	1.04(0.79–1.36)	1.09(0.83–1.43)
Inpatient hospital days zero	Reference	0.89(0.67–1.19)	0.80(0.59–1.10)	1.03(0.70–1.50)	0.57(0.39–0.83)

Note: Based on zero-inflated negative binomial regression

^†^Model 2 was adjusted for age, sex, marital status, area of residence, education, sleeping time.

^∥^Model 5 was adjusted for age, sex, marital status, area of residence, education, sleeping time, smoke status, drink status, chronic diseases, socioeconomic status, health insurance, and economic development regions.

**Fig. 2:**
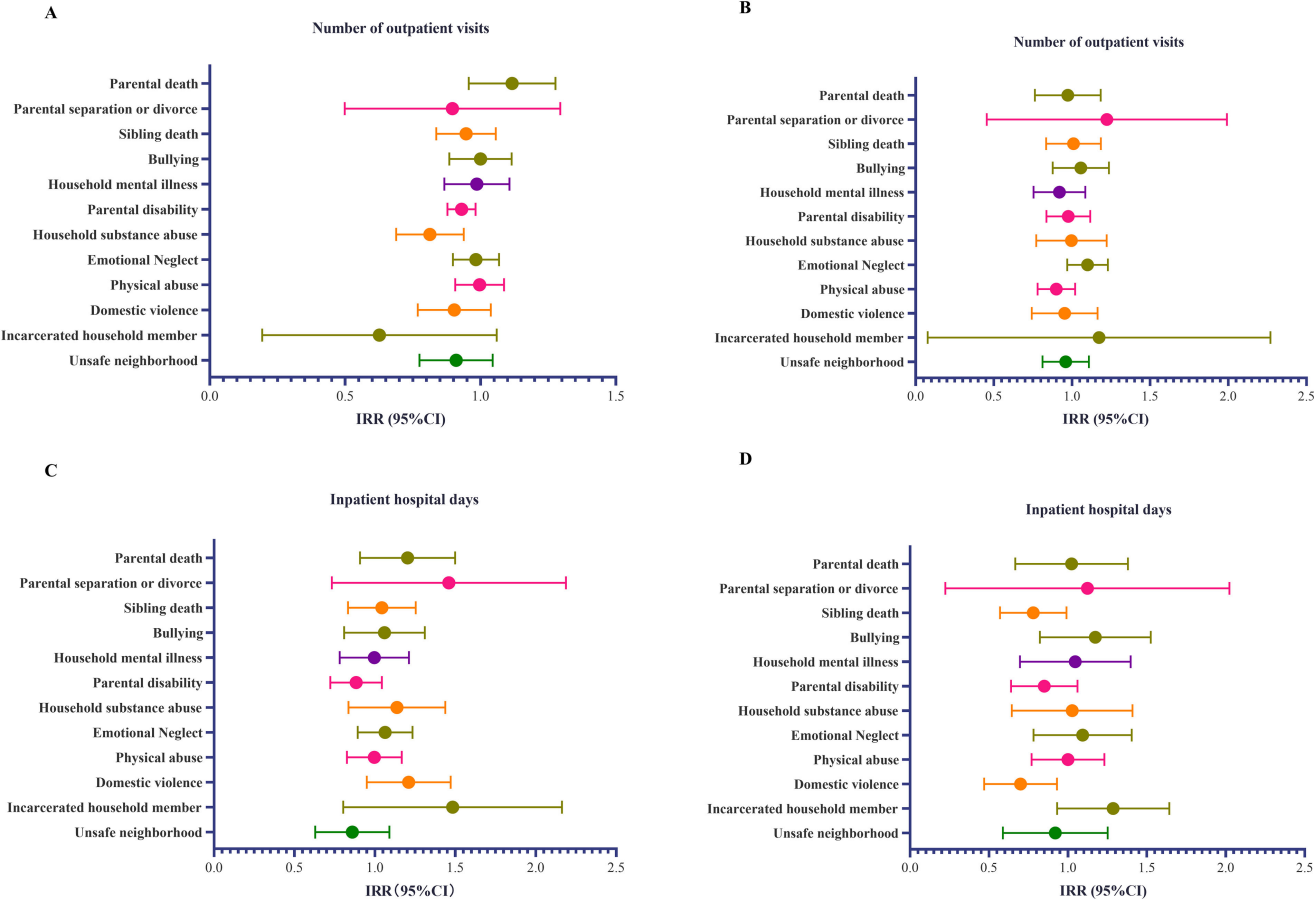
Association between the adverse childhood experience indicator and the three outcomes. Legend: ACE, adverse childhood experience; IRR, incidence rate ratio; OR, odds ratio. The model was adjusted for age, sex, marital status, area of residence, education, sleeping time, smoking status, drink status, chronic diseases, socioeconomic status, health insurance, and economic development regions. A, Association between the adverse childhood experience indicator and the number of outpatient visits. B, Association between the adverse childhood experience indicator and the inpatient hospital days. C, Association between the adverse childhood experience indicator and the catastrophic health expenditures.

**Fig. 3:**
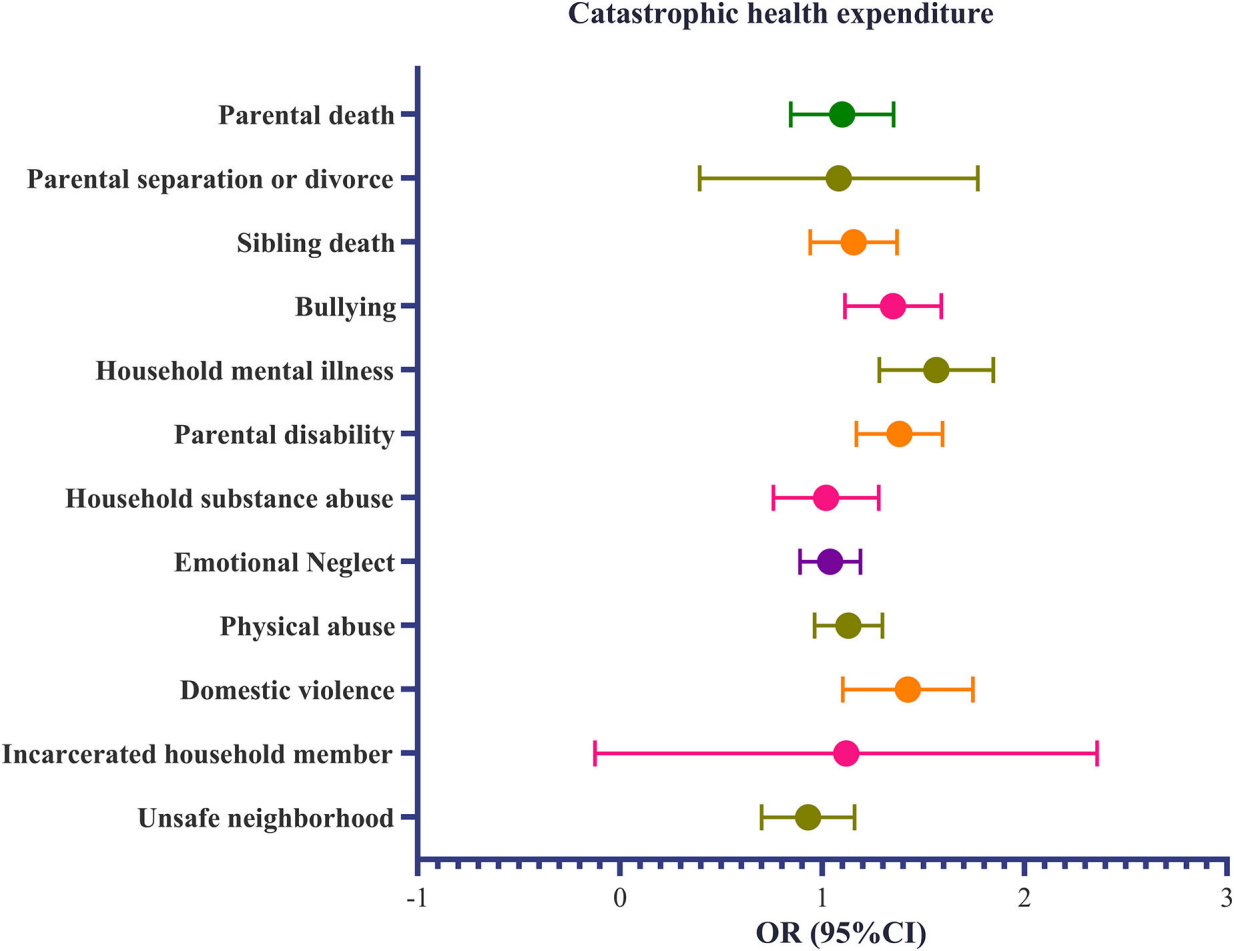
Association between the adverse childhood experience indicator and catastrophic health expenditure.

When we examined the ACE indicators, we found that, under the assumption that the observed values are not always zero, household substance abuse was associated with fewer outpatient visits (IRR: 0.81, 95%CI: 0.69–0.94; Panel A of Fig. 2). We found that domestic violence (OR = 0.68, 95% CI: 0.48–0.94; Panel D of Fig. 2) reduced the proportion of patients in the “non-hospitalized group” by 32%. Participants who experienced bullying, household mental illness, parental disability, and domestic violence were 34%, 55%, 37%, and 40% more likely to have CHE compared to those who did not experience any ACEs (bullying: OR = 1.34, 95%CI: 1.12–1.59; household mental illness: OR = 1.55, 95% CI: 1.29–1.85; parental disability: OR = 1.37, 95%CI: 1.17–1.60; domestic violence: OR = 1.40, 95%CI: 1.11–1.75; Fig. 3).

Mediating analysis was conducted to examine the role of chronic diseases in the relationship between ACEs and the number of outpatient visits, inpatient hospital days, and CHE. The average causal mediation effects (ACME) and average direct effects (ADE) are presented in Table 4. Chronic diseases served as a mediating factor between ACEs and CHE (ACME = 0.000904, P = 0.03; ADE = 0.00813, P < 0.001). The results of the analysis of chronic diseases as a binary variable are similar (Table S8). The sensitivity analysis of the mediator showed that the estimated effect of the mediator without ACE was relatively sensitive to unmeasured confounding factors (Rho = 0.12) (Fig. S3).

**Table 4:** Mediating effect of chronic diseases on the association between adverse childhood experiences and three outcomes

**Dependent variable**	**ACME**	**P value**	**ADE**	**P value**	**Prop. Mediated**	**P value**
Number of outpatient visits	0.00314	0.61	−0.10915	0.24	−0.02964	0.75
Inpatient hospital days	0.001505	0.152	0.006571	0.078	0.186367	0.162
Catastrophic health expenditure	0.000904	0.03	0.00813	<0.001	0.100	0.03

Note: ACE, adverse childhood experience; ACME, average causal mediation effects (indirect effect); ADE, average direct effects; Prop. Mediated, proportion mediation.

Notably, the number of chronic diseases reached three or more, the proportion of outpatient visits declined, while the proportion of inpatient hospitalizations rose (Fig. S2).

## Discussion

As the number of ACEs increased, the likelihood of CHE also increased. However, no significant association was observed between ACEs and the number of outpatient visits or inpatient hospital days. These findings suggest that the direct impact of ACEs on the financial burden of healthcare is more pronounced than directly leading to more frequent medical visits. ACEs are associated with socioeconomic status, and lower socioeconomic status may restrict an individual’s access to medical resources, leading to reliance on high-cost medical services during times of urgent need. Addressing the long-term health effects of ACEs is essential for reducing healthcare costs and improving patient outcomes [22–24].

Several studies suggest that individuals with a greater number of ACEs may struggle to form and maintain healthy social relationships [25–27]. This social dysfunction resulting from ACEs can lead to various health-compromising behaviors, such as cigarette use [28], thereby increasing the likelihood of developing chronic conditions and necessitating more frequent medical care [29–32]. Additionally, Senaratne et al. conducted a meta-analysis revealing that the odds of multimorbidity increased by 12.9% for each additional exposure to ACEs [33]. This increased risk of multimorbidity further elevates the likelihood of seeking medical care.

Negative psychological issues resulting from ACEs have also been identified [34]. These issues often necessitate greater psychological counseling and assessment. However, such counseling is typically not covered by medical insurance for chronic diseases in China, leading to significant out-of-pocket medical expenses. In recent years, the Chinese government has undertaken ongoing efforts to promote psychological health, enhance mental health literacy, optimize the allocation of limited mental health resources, and strengthen interventions for common mental disorders through its Healthy China 2030 Plan [35].

Our research findings indicate that having experienced more adverse events during childhood is related to the medical burden of middle-aged and elderly adults. Despite the inconsistencies in the ACE assessment, we considered multiple ACEs whenever possible. We further explored the mediating role of chronic diseases to provide further insights.

Like other retrospective studies, this research has several limitations. First, respondents may inaccurately report their experiences or behaviors. Second, due to the design of the questionnaire, the study only included female guardians in the assessment of emotional neglect, thereby excluding the potential influence of male guardians. Third, this study may underestimate the effects of chronic diseases (e.g., infectious diseases) by including only a limited number of non-communicable chronic diseases as covariates in the database. Fourth, we excluded over 7,000 individuals from an initial pool of 18,775 eligible participants, accounting for approximately 40% of the sample. This exclusion raises important questions about the potential impact on our estimates and the overall results of the study. The characteristics of the excluded participants may differ from those included in the analysis, potentially limiting the generalizability of our findings. Fifth, our study derived annual outpatient expenses by annualizing monthly reports (monthly out-of-pocket × 12), a simplifying assumption that may not capture seasonal variations or month-to-month changes in actual healthcare use. While sensitivity analyses bolstered the robustness of our findings, a degree of measurement error inherent to this method persists. Sixth, the mediating path in the control group of the study showed relatively low robustness. Although we tried our best to control for known confounding variables, this result indicates that the existence of an unknown confounding factor with moderate explanatory power could challenge the current conclusion. Seventh, although the weighted scoring method has the potential to enhance measurement accuracy, its evaluation was not feasible owing to the unavailability of requisite data. Future research should prioritize the design of specialized prospective cohort studies to further validate the potential causal relationship. Finally, for reasons of feasibility and reproducibility, only healthcare data from 2015 were used, which may not capture changes and trends in subsequent years.

## Conclusion

ACE has the capacity to predict CHE, and the findings of this study reinforce the potential pathway through which ACE may exert its influence on CHE via the burden of chronic diseases. Furthermore, the mediation analysis conducted in this study highlights the significant mediating role of chronic diseases in the relationship between ACE and CHE. This cross-sectional study examining the associations of ACEs with medical-seeking behavior and healthcare expenditures is valuable for understanding how ACEs affect healthcare utilization and physical health. Measures should be implemented to prevent ACEs to lessen the economic burden on individuals and families as well as the adverse impact of national financial risk [36].

## Abbreviations

CHARLS: China Health and Retirement Longitudinal Study
SDs: Standard Deviations
ACEs: Adverse Childhood Experiences
CHEs: Catastrophic Health Expenditures
ORs: Odds Ratios
CIs: Confidence Intervals
IRRs: Incidence Rate Ratios

## Supplementary information

**Additional file 1:** **Table S1** Questionnaire items of each adverse childhood experience indicator. **Table S2** Questionnaire information related to covariates. **Table S3** Baseline missing values for 11,072 Chinese adults. **Table S4** Prevalence of adverse childhood experience indicator. **Table S5** The association between adverse childhood experiences and the number of outpatient visits as well as the number of days of hospitalization based on zero-inflated negative binomial regression. **Table S6** Association of adverse childhood experiences with the number of outpatient visits and inpatient hospital days (Poisson regression analysis). **Table S7** Association of adverse childhood experiences with number of outpatient visits, inpatient hospital days, and catastrophic health expenditure with other imputed data sets. **Table S8.** Mediating effect of chronic diseases on the association between adverse childhood experiences and three outcomes. **Fig. S1** Prevalence of non-zero outpatient visits, non-zero inpatient hospital days, and catastrophic health expenditure by age. **Fig. S2** Prevalence of non-zero outpatient visits, non-zero inpatient hospital days, and catastrophic health expenditure by chronic diseases. **Fig. S3** sensitivity analysis of the mediator.

## Declarations

### Availability of data and material

The data that support the findings of this study are available from CHARLS project site, subject to registration and application process. Further details can be found at https://charls.charlsdata.com/pages/Data/
