# Supplementary material for: Association of adverse childhood experiences with health service use and catastrophic health expenditures in China: evidence from the China Health and Retirement Longitudinal Study
Source: Environ Health Prev Med. 2026 Jan 1;31:1. doi: 10.1265/ehpm.25-00012 (PMC12783321; doi:10.1265/ehpm.25-00012)
Supplement: Supplementary file 1 — Additional file 1: Table S1 Questionnaire items of each adverse childhood experience indicator. Table S2 Questionnaire information related to covariates. Table S3 Baseline missing values for 11,072 Chinese adults. Table S4 Prevalence of adverse childhood experience indicator. Table S5 The association between adverse childhood experiences and the number of outpatient visits as well as the number of days of hospitalization based on zero-inflated negative binomial regression. Table S6 Association of adverse childhood experiences with the number of outpatient visits and inpatient hospital days (Poisson regression analysis). Table S7 Association of adverse childhood experiences with number of outpatient visits, inpatient hospital days, and catastrophic health expenditure with other imputed data sets. Table S8. Mediating effect of chronic diseases on the association between adverse childhood experiences and three outcomes. Fig. S1 Prevalence of non-zero outpatient visits, non-zero inpatient hospital days, and catastrophic health expenditure by age. Fig. S2 Prevalence of non-zero outpatient visits, non-zero inpatient hospital days, and catastrophic health expenditure by chronic diseases. Fig. S3 sensitivity analysis of the mediator. [file ehpm-31-001-s001.docx]

Supplement

Table S1 Questionnaire items of each adverse childhood experience indicator.

Table S2 Questionnaire information related to covariates.

Table S3 Baseline missing values for 11,072 Chinese adults.

Table S4 Prevalence of adverse childhood experience indicator.

Table S5 The association between adverse childhood experiences and the number of outpatient visits as well as the number of days of hospitalization based on zero-inflated negative binomial regression.

Table S6 Association of adverse childhood experiences with the number of outpatient visits and inpatient hospital days (Poisson regression analysis).

Table S7 Association of adverse childhood experiences with number of outpatient visits, inpatient hospital days, and catastrophic health expenditure with other imputed data sets.

Table S8. Mediating effect of chronic diseases on the association between adverse childhood experiences and three outcomes.

Fig. S1 Prevalence of non-zero outpatient visits, non-zero inpatient hospital days, and catastrophic health expenditure by age.

Fig. S2 Prevalence of non-zero outpatient visits, non-zero inpatient hospital days, and catastrophic health expenditure by chronic diseases.

Fig. S3 sensitivity analysis of the mediator.

Table S1 Questionnaire items of each adverse childhood experience indicator.

| ACE indicators | Questionnaire Items | Information |
| --- | --- | --- |
| Parental death | Is your biological mother alive? | Based on these questions and the age of the respondent, whether the respondent's parents died before the respondent was 17 years old was calculated  (Yes=1 or No=0) |
|  | In what year did she pass away or how old was she then? |  |
|  | What was your biological mother’s birth year? |  |
|  | Is your biological father alive? |  |
|  | What was your biological father’s birth year? |  |
|  | In what year did he pass away or how old was he then? |  |
| Parental separation or divorce | Were your biological parents divorced? | Whether the respondent's parents' separation or divorce occurred before the respondent was 17 years old (Yes=1 or No=0) |
|  | What was your age when your parents divorce? |  |
| Bullying | When you were a child, how often were you picked on or bullied by kids in your neighborhood? Is it often, sometimes, rarely or never? | often/sometimes=1  not very often/never=0 |
|  | When you were a child, how often were you picked on or bullied by kids in your school? Is it often, sometimes, rarely or never? |  |
| Household mental illness | Did your female guardian have abnormality of mind when you were young? | Yes=1 or No=0 |
|  | Did your male guardian have abnormality of mind when you were young? |  |
|  | Was this problem of your female guardian, sadness or depression during all, most, some, or only a little of your childhood? | during all/most=1  some/only a little of the childhood=0 |
|  | Was this problem of your male guardian, sadness or depression during all, most,  some, or only a little of your childhood? |  |
| Parental disability | Did your female guardian have a long time be sick on bed when you were young? | yes=1 or no=0 |
|  | Did your female guardian have a serious deformity when you were young? |  |
|  | Did your female guardian have a serious deformity when you were young? |  |
|  | Did your male guardian have a serious deformity when you were young? |  |
| Emotional neglect | How much love and affection did your female guardian give you while you were growing up? | often/sometimes=0  rarely/never=1 |
|  | How much effort did your female guardian put into watching over you? | a lot/some=0  a little/not at all=1 |
| Physical abuse | When you were growing up, did your female guardian ever hit you? Was that often, sometimes, rarely, or never? | often/sometimes=1  rarely/never=0 |
|  | When you were growing up, did your male guardian ever hit you? Was that often, sometimes, rarely, or never? |  |
| Domestic violence | Have your father ever beat up your mother? | often/sometimes=1  not very often/never=0 |
|  | Have your mother ever beat up your father? |  |
| Incarcerated household member | During the years you were growing up, did your female/male guardian ever been arrested or sent to prison? | yes=1 or no=0 |
| Household substance abuse | During the years you were growing up, did your female/male guardian ever have alcoholism or/and drug? | yes=1 or no=0 |
| Unsafe neighborhood | When you were a child, how often were you picked on or bullied by kids in your neighborhood? Is it often, sometimes, rarely or never? | often/sometimes=1  not very often/never=0 |
| Sibling death | Any of the siblings was dead before participant was 17 years. | yes=1 or no=0 |

Table S2 Questionnaire information related to covariates.

| Covariate indicators | Questionnaire Items | Information | |
| --- | --- | --- | --- |
| age | What’s your actual date of birth? |  | |
| sex | Interviewer record R’s gender. | Male  Female | |
| marital status | What is your marital status? | Married | 1.Married with spouse present  2.Married but not living with spouse temporarily for reasons such as work |
|  |  | Unmarried | 3. Separated  4. Divorced  5. Widowed  6. Never married  7. Cohabitated |
| education level | Have your highest level of education changed from last wave? If so, what’s the highest level of education your have attained now?(not including adult education) | None | 1. No formal education (illiterate) |
|  |  | Home school to primary school | 2. Did not finish primary school  3. Sishu/home school  4. Elementary school |
|  |  | Middle school or above | 5. Middle school  6. High school  7. Vocational school  8. Two-/Three-Year College/Associate degree  9. Four-Year College/Bachelor’s degree  10. Master’s degree  11. Doctoral degree/Ph.D.  12. No changing |
| smoking status | In last interview，did you have the habit or have you totally quit? | Smoker | 1. Still have |
|  |  | Ever smoker | 2. Quit |
|  |  | Never smoking | 3. Never smoked |
| drinking habits | Did you ever drink alcoholic beverages in the past? How often? | Never drinking | I never had a drink |
|  |  | Less than once a month | I used to drink less than once a month. |
|  |  | More than once a month | I used to drink more than once a month. |
| place of residence | Was it village or city/town? | Urban | 1. Main city zone  2. Combination zone between urban and rural areas  3. The town center |
|  |  | Rural | 4. ZhenXiang area  5. special area  6. Township central  7. Village |
| number of chronic diseases | Have you been diagnosed with [conditions listed below, read one by one] by a doctor? | 1. Hypertension  2. Dyslipidemia (elevation of low density lipoprotein, triglycerides (TGs), and total cholesterol, or a low high density lipoprotein level)  3. Diabetes or high blood sugar  4. Cancer or malignant tumor (excluding minor skin cancers)  5. Chronic lung diseases, such as chronic bronchitis, emphysema (excluding tumors, or cancer)  6. Liver disease (except fatty liver, tumors, and cancer)  7. Heart attack, coronary heart disease, angina, congestive heart failure, or other heart problems  8. Stroke  9. Kidney disease (except for tumor or cancer)  10. Stomach or other digestive disease (except for tumor or cancer)  11. Emotional, nervous, or psychiatric problems  12. Memory-related disease  13. Arthritis or rheumatism  14. Asthma | |
| economic development region | What’s your address now? | Five economic development regions were designated according to the ranking of China’s provincial gross domestic product per capita in 2015 (Group 1, >$12,000; Group 2, $12,000 to >$10,000; Group 3, $10,000 to >$7,000; Group 4, $7,000 to >$6,000; and Group 5, ≤$6,000). | |
| socioeconomic group | In the past week, how much did your household spend on food (excluding eating out expenditure, alcohol, cigarettes, cigars and tobacco expenditure)? | ____ Yuan | |
|  | In the past week, what was the market value of the food that members of the household consumed that you grew yourselves? | ____ Yuan | |
|  | Amongit, how much did your household spend on eating out? | ____ Yuan | |
|  | Among it, how much did your household spend on alcohol, Cigarettes, cigars and tobacco? | ____ Yuan | |
|  | Please tell me the expenditure last month for your household for the following items. | 1. Communication fees (including post, internet usage, telephone and cell phone usage) ____Yuan  2. Utilities: Water and electricity ____ Yuan  3. Fuels (including gas, coal, etc.) ____ Yuan  4. Fees for Matron, housekeepers and servants __Yuan  5. Local Transportation ____ Yuan  6. Household items and personal toiletries that are used daily plus beauty treat ments (e.g., detergent, soap, toothpaste, toothbrush, cosmetics, beauty salon, etc.) ____Yuan  7. Entertainment (including fees to buy books, newspapers, VCCs, DVDs, going to cinema and bars) ____Yuan | |
|  | In the last year how much did your household spend on the following items? | 1. Clothing and bedding ____ Yuan  2. Long distance traveling expenses ____ Yuan  3. Heating (centrally heated) ____ Yuan  4. Furniture, consumption of durable goods and electronics, includes refrigerator, washing machine, TV, computers and expensive instruments like pianio. ____ Yuan  5. Education and training (including tuition, training fees, etc.) ____ Yuan  6. Medical expenditure ____ Yuan  7. Fitness expenditures ____ Yuan  8. Beauty (including make-ups, facials, massages, etc.) ____ Yuan  9. Automobiles ____ Yuan  10. Purchase, Maintenance and repair (of transportation vehicles, appliances, communication products, etc.) ____Yuan  11. Property management fees (including parking fee) ____ Yuan  12. Taxes and fees turned over to the government ____ Yuan  13. Donations to the society (including cash, and items like food, clothing, etc.) ____ Yuan | |
| health insurance | Are you the policy holder/primary beneficiary of any of the types of health insurance listed below? (circle all that apply) | UEBMI | 1. Urban employee medical insurance (yi-bao) |
|  |  | URBMI | 2. Urban resident medical insurance |
|  |  | NRCMS | 3. New cooperative medical insurance (he-zuo-yi-liao) |
|  |  | Others | 4. Urban and rural resident medical insurance  5. Government medical insurance (gong-fei)  6. Medical aid  7. Private medical insurance: purchased by work unit  8. Private medical insurance: purchased by individual  9. Urban non-employed persons’s health insurance  10. Other medical insurance (specify) |
|  |  | None | 11. No insurance |
| sleep duration | During the past month, how many hours of actual sleep did you get at night (average hours for one night)? (This may be shorter than the number of hours you spend in bed.) | ____ hours | |

Table S3 Baseline missing values for 11,072 Chinese adults.

| Characteristics | ACE=0 (n=2748) | ACE=1 (n=3484) | ACE=2（n=2479） | ACE=3 (n=1396) | ACE≥4 (n=965) |
| --- | --- | --- | --- | --- | --- |
| Residence | 4 | 13 | 4 | 7 | 4 |
| Educational level | 3 |  |  |  |  |
| Sleeping time | 1 |  | 1 |  |  |
| Drinking status | 668 | 992 | 760 | 410 | 293 |
| Smoking status | 1633 | 1896 | 1301 | 741 | 524 |

Note: ACE, adverse childhood experience.

Table S4 Prevalence of adverse childhood experience indicator.

| **Type of ACEs** | Prevalence (%) |
| --- | --- |
| **Parental death** | 8.02% |
| **Parental separation or divorce** | 0.83% |
| **Sibling death** | 13.36% |
| **Bullying** | 15.33% |
| **Household mental illness** | 12.01% |
| **Parental disability** | 20.72% |
| **Household substance abuse** | 7.41% |
| **Emotional Neglect** | 31.50% |
| **Physical abuse** | 28.40% |
| **Domestic violence** | 7.41% |
| **Incarcerated household member** | 0.27% |
| **Unsafe neighborhood** | 7.96% |

Note: ACE, adverse childhood experience.

Table S5. The association between adverse childhood experiences and the number of outpatient visits as well as the number of days of hospitalization based on zero-inflated negative binomial regression

|  | ACE=0 | ACE=1 | ACE=2 | ACE=3 | ACE≥4 |  |
| --- | --- | --- | --- | --- | --- | --- |
|  |  | IRR(95%CI) | IRR(95%CI) | IRR(95%CI) | IRR(95%CI) |  |
| Model1^a^ | | | | | | |
| Number of outpatient visits count | Reference | 1.01(0.91-1.13) | 0.88(0.78-0.99) | 0.92(0.80-1.05) | 0.88(0.75-1.03) |  |
| Number of outpatient visits zero | Reference | 1.02(0.87-1.19) | 1.05(0.88-1.25) | 0.99(0.81-1.21) | 0.97(0.77-1.21) |  |
| Inpatient hospital days count | Reference | 0.99 (0.81-1.20) | 0.87 (0.70-1.09) | 1.03 (0.80-1.32) | 0.99 (0.78-1.26) |  |
| Inpatient hospital days zero | Reference | 0.88(0.67-1.17) | 0.81(0.6-1.09) | 0.96(0.67-1.38) | 0.54(0.38-0.76) |  |
| Model2^b^ | | | | | | |
| Number of outpatient visits count | Reference | 0.99(0.89-1.11) | 0.86(0.77-0.97) | 0.89(0.78-1.03) | 0.85(0.73-0.99) |  |
| Number of outpatient visits zero | Reference | 1.02(0.87-1.19) | 1.05(0.88-1.25) | 0.98(0.80-1.20) | 0.96(0.76-1.21) |  |
| Inpatient hospital days count | Reference | 0.99(0.81-1.23) | 0.87(0.70-1.10) | 1.01(0.78-1.32) | 1.02(0.79-1.31) |  |
| Inpatient hospital days zero | Reference | 0.87(0.67-1.15) | 0.78(0.58-1.05) | 0.92(0.64-1.32) | 0.52(0.36-0.73) |  |
| Model3^c^ | | | | | | |
| Number of outpatient visits count | Reference | 0.99(0.89-1.10) | 0.87(0.78-0.98) | 0.90(0.78-1.04) | 0.86(0.74-1.01) |  |
| Number of outpatient visits zero | Reference | 1.01(0.87-1.19) | 1.05(0.88-1.24) | 0.97(0.79-1.19) | 0.96(0.76-1.21) |  |
| Inpatient hospital days count | Reference | 1.05(085-1.29) | 0.87(0.69-1.10) | 1.08(0.83-1.40) | 1.09(0.84-1.41) |  |
| Inpatient hospital days zero | Reference | 0.89(0.67-1.18) | 0.77(0.57-1.05) | 0.95(0.66-1.37) | 0.54(0.38-0.77) |  |
| Model4^d^ | | | | | | |
| Number of outpatient visits count | Reference | 0.99(0.89-1.10) | 0.88(0.78-0.99) | 0.91(0.79-1.05) | 0.87(0.75-1.02) |  |
| Number of outpatient visits zero | Reference | 1.01(0.87-1.19) | 1.05(0.88-1.25) | 0.98(0.80-1.21) | 0.97(0.77-1.22) |  |
| Inpatient hospital days count | Reference | 1.04(0.85-1.29) | 0.87(0.69-1.09) | 1.07(0.82-1.40) | 1.08(0.82-1.40) |  |
| Inpatient hospital days zero | Reference | 0.90(0.85-1.29) | 0.79(0.69-1.09) | 0.98(0.82-1.40) | 0.56(0.83-1.41) |  |
| Model5^e^ | | | | | | |
| Number of outpatient visits count | Reference | 0.99(0.89-1.10) | 0.89(0.79-0.99)* | 0.92(0.80-1.06) | 0.89(0.76-1.04) |  |
| Number of outpatient visits zero | Reference | 1.01(0.86-1.18) | 1.04(0.88-1.24) | 0.97(0.79-1.19) | 0.96(0.76-1.21) |  |
| Inpatient hospital days count | Reference | 0.97(0.87-1.21) | 0.84(0.67-1.07) | 1.04(0.79-1.36) | 1.09(0.83-1.43) | 1 |
| Inpatient hospital days zero | Reference | 0.89(0.67-1.19) | 0.80(0.59-1.10) | 1.03(0.70-1.50) | 0.57(0.39-0.83) |  |

Note: ACEs, Adverse Childhood Experience; IRR, Adjusted Incidence Rate Ratio; OR, odds ratios.

^a^Model1 was the cruel model

^b^Model2 was adjusted for age, sex, marital status, area of residence, education, sleeping time.

^c^Model3 was adjusted for age, sex, marital status, area of residence, education, sleeping time, smoking status, drink status.

^d^Model4 was adjusted for age, sex, marital status, area of residence, education, sleeping time, smoking status, drink status, chronic diseases.

^e^Model5 was adjusted for age, sex, marital status, area of residence, education, smoking status, drink status, socioeconomic status, health insurance, sleeping time, chronic diseases, and economic development regions.

Table S6 Association of adverse childhood experiences with the number of outpatient visits and inpatient hospital days (Poisson regression analysis).

|  | **ACE=0** | **ACE=1** | **ACE=2** | **ACE=3** | **ACE≥4** | **P-trend** |
| --- | --- | --- | --- | --- | --- | --- |
| **Model1^a^** |  | IRR (95%CI) | IRR (95%CI) | IRR (95%CI) | IRR (95%CI) |  |
| **Number of outpatient visits** | Reference | 1.00(0.97,1.03) | 0.84(0.81,0.87) | **0.93(0.90,0.96)^f^** | **0.91(0.87,0.94)** | **<0.001** |
| **Inpatient hospital days** | Reference | 1.11 (0.94,1.32) | 1.07 (0.89,1.29) | 1.07 (0.86,1.33) | **1.78(1.44,2.19)** | **<0.001** |
| **Model2^b^** |  |  |  |  |  |  |
| **Number of outpatient visits** | Reference | **0.99(0.96,1.02)** | **0.83(0.80,0.85)** | **0.91(0.88,0.95)** | **0.89(0.85,0.93)** | **<0.001** |
| **Inpatient hospital days** | Reference | 1.14(0.96,1.35) | 1.11(0.93,1.34) | 1.12(0.90,1.39) | **1.88(1.518,2.31)** | **<0.001** |
| **Model3^c^** |  |  |  |  |  |  |
| **Number of outpatient visits** | Reference | **1.00(0.97,1.02)** | **0.84(0.81,0.86)** | **0.93(0.99,0.97)** | **0.90(0.860,0.94)** | **<0.001** |
| **Inpatient hospital days** | Reference | 1.13 (0.96,1.34) | 1.13 (0.94,1.36) | 1.11(0.888,1.376) | **1.83(1.477,2.25)** | **<0.001** |
| **Model4^d^** |  |  |  |  |  |  |
| **Number of outpatient visits** | Reference | **1.00(0.97,1.02)** | **0.84(0.81,0.86)** | **0.93(0.90,0.97)** | **0.90(0.862,0.94)** | **<0.001** |
| **Inpatient hospital days** | Reference | 1.11 (0.94,1.32) | 1.09(0.90,1.31) | 1.07(0.86,1.33) | **1.74(1.41,2.14)** | **<0.001** |
| **Model5^e^** |  |  |  |  |  |  |
| **Number of outpatient visits** | Reference | **1.00(0.97,1.03)** | **0.84(0.82,0.87)** | **0.94(0.91,0.98)** | **0.91(0.87,0.95)** | **<0.001** |
| **Inpatient hospital days** | Reference | 1.09(0.92,1.30) | 1.058(0.88,1.27) | 1.006(0.81,1.25) | **1.692(1.37,2.09)** | **<0.001** |

Note: ACEs, Adverse Childhood Experience; CI, confidence interval; IRR, Adjusted Incidence Rate Ratio.

^a^Model1 was the cruel model.

^b^Model2 was adjusted for age, sex, marital status, area of residence, education, sleeping time.

^c^Model3 was adjusted for age, sex, marital status, area of residence, education, sleeping time, smoking status, drink status.

^d^Model4 was adjusted for age, sex, marital status, area of residence, education, sleeping time, smoking status, drink status, chronic diseases.

^e^Model5 was adjusted for age, sex, marital status, area of residence, education, smoking status, drink status, socioeconomic status, health insurance, sleeping time, chronic diseases, and economic development regions.

^f^Bold IRR [95% CI] indicates statistical significance.

Table S7 Association of adverse childhood experiences with number of outpatient visits, inpatient hospital days, and catastrophic health expenditure with other imputed data sets.

|  | ACE=0 | ACE=1 | ACE=2 | ACE=3 | ACE≥4 |  |
| --- | --- | --- | --- | --- | --- | --- |
|  |  | IRR/OR(95%CI) | IRR/OR(95%CI) | IRR/OR(95%CI) | IRR/OR(95%CI) |  |
| Imputed data 1 | | | | | | |
| Number of outpatient visits (count) | Reference | 1.01(0.91-1.12) | 0.91(0.81-1.02) | 0.91(0.79-1.05) | 0.88(0.76-1.03) |  |
| Number of outpatient visits (zero) | Reference | 1.01(0.86-1.18) | 1.05(0.88-1.24) | 0.98(0.80-1.20) | 0.96(0.76-1.21) |  |
| Inpatient hospital days (count) | Reference | 0.96(0.77-1.20) | 0.85(0.67-1.07) | 1.03(0.78-1.35) | 1.06(0.81-1.39) |  |
| Inpatient hospital days (zero) |  | 0.87(0.65-1.17) | 0.79(0.58-1.09) | 0.99(0.68-1.46) | 0.56(0.39-0.81) |  |
| Catastrophic health expenditure | Reference | 1.06(0.87,1.29) | 1.27(1.04,1.56) | 1.35(1.07,1.70) | 1.76(1.37,2.24) |  |
| Imputed data 2 | | | | | | |
| Number of outpatient visits (count) | Reference | 1.00(0.90-1.11) | 0.88(0.78-0.99) | 0.90(0.79-1.04) | 0.87(0.75-1.02) |  |
| Number of outpatient visits (zero) | Reference | 1.01(0.86-1.19) | 1.04(0.87-1.24) | 0.97(0.79-1.19) | 0.96(0.76-1.01) |  |
| Inpatient hospital days (count) | Reference | 0.97(0.78-1.21) | 0.87(0.69-1.09) | 1.01(0.76-1.33) | 1.04(0.79-1.37) |  |
| Inpatient hospital days (zero) | Reference | 0.88(0.66-1.18) | 0.78(0.57-1.08) | 1.00(0.69-1.47) | 0.57(0.40-0.82) |  |
| Catastrophic health expenditure | Reference | 1.07(0.88,1.30) | 1.27(1.04,1.56) | 1.36(1.08,1.71) | 1.79(1.40,2.28) |  |
| Imputed data 3 | | | | | | |
| Number of outpatient visits (count) | Reference | 1.00(0.90-1.12) | 0.89(0.79-1.00) | 0.91(0.79-1.05) | 0.88(0.75-1.03) |  |
| Number of outpatient visits (zero) | Reference | 1.01(0.86-1.19) | 1.05(0.88-1.25) | 0.98(0.80-1.20) | 0.96(0.76-1.21) |  |
| Inpatient hospital days (count) | Reference | 0.94(0.76-1.17) | 0.83(0.65-1.05) | 0.97(0.73-1.26) | 1.03(0.79-1.35) |  |
| Inpatient hospital days (zero) |  | 0.89(0.67-1.19) | 0.76(0.55-1.06) | 0.99(0.68-1.46) | 0.58(0.41-0.83) |  |
| Catastrophic health expenditure | Reference | 1.05(0.87,1.27) | 1.25(1.02,1.53) | 1.33(1.06,1.68) | 1.750(1.37,2.23) |  |
| Imputed data 4 | | | | | | |
| Number of outpatient visits (count) | Reference | 1.00(0.90-1.12) | 0.89(0.79-0.99) | 0.92(0.80-1.06) | 0.88(0.75-1.02) |  |
| Number of outpatient visits (zero) | Reference | 1.02(0.87-1.19) | 1.05(0.88-1.25) | 0.98(0.80-1.20) | 0.96(0.76-1.21) |  |
| Inpatient hospital days (count) | Reference | 0.93(0.75-1.16) | 0.84(0.66-1.06) | 0.97(0.74-1.27) | 1.02(0.78-1.34) |  |
| Inpatient hospital days (zero) |  | 0.86(0.65-1.16) | 0.79(0.58-1.09) | 0.97(0.67-1.42) | 0.55(0.38-0.79) |  |
| Catastrophic health expenditure | Reference | 1.05(0.86,1.27) | 1.25(1.02,1.53) | 1.33(1.06,1.68) | 1.74(1.36,2.22) |  |

Note: ACEs, Adverse Childhood Experience; CI, confidence interval; IRR, Adjusted Incidence Rate Ratio; OR, odds ratio. The model was adjusted for age, sex, marital status, area of residence, education, sleeping time, smoking status, drink status, chronic diseases, socioeconomic status, health insurance, and economic development regions.

Table S8. Mediating effect of chronic diseases on the association between adverse childhood experiences and three outcomes

| **Dependent variable**  **(binary variable)** | **ACME** | **P value** | **ADE** | **P value** | **Prop. Mediated** | **P value** |
| --- | --- | --- | --- | --- | --- | --- |
| Number of outpatient visits | 0.00332 | 0.34 | -0.11277 | 0.23 | -0.03034 | 0.49 |
| Inpatient hospital days | 0.000685 | 0.052 | 0.00896 | 0.014 | 0.071 | 0.060 |
| Catastrophic health expenditure | 0.000452 | 0.004 | 0.008350 | <0.001 | 0.051316 | 0.004 |

Note: ACE, adverse childhood experience; ACME, average causal mediation effects (indirect effect); ADE, average direct effects; Prop. Mediated, proportion mediation.

Fig. S1 Prevalence of non-zero outpatient visits, non-zero inpatient hospital days, and catastrophic health expenditure by age.

Fig. S2 Prevalence of non-zero outpatient visits, non-zero inpatient hospital days and catastrophic health expenditure by chronic diseases.

Note: ACEs, Adverse Childhood Experiences.


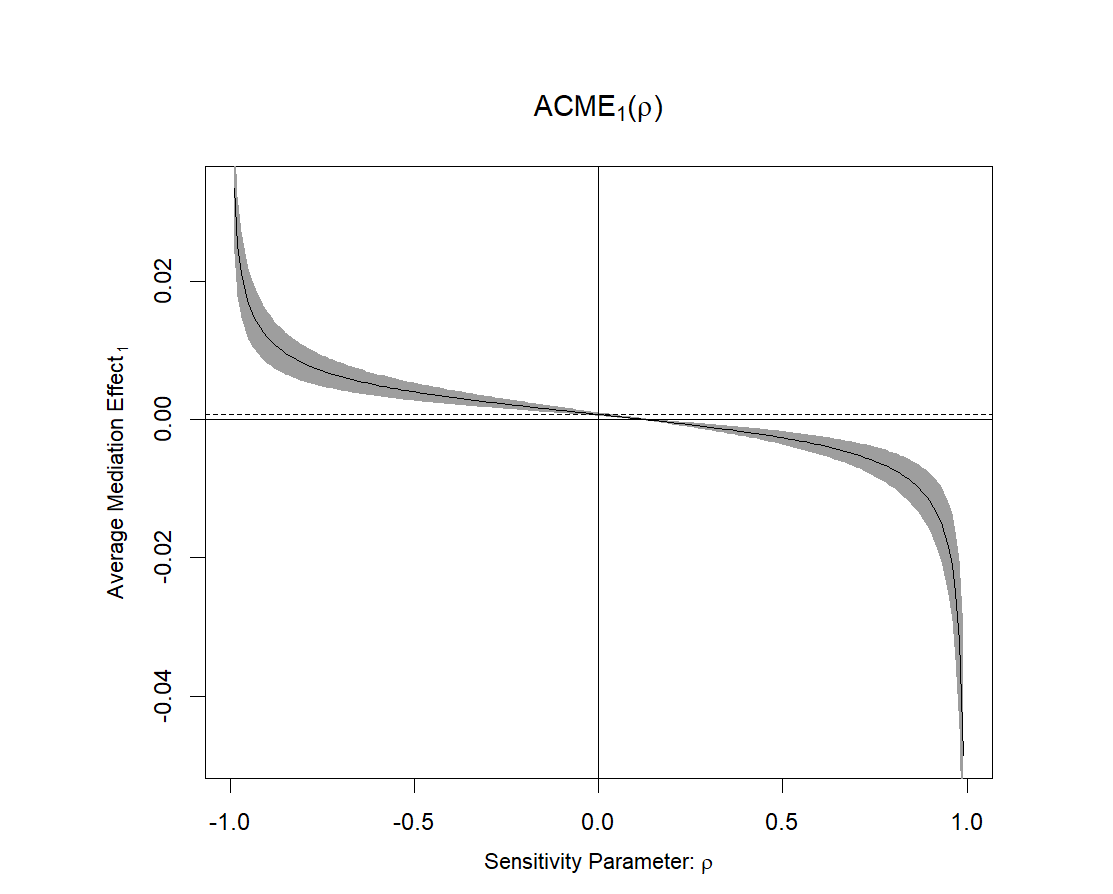
Fig. S3. sensitivity analysis of the mediator
